# Married women’s decision-making autonomy on modern contraceptive use and its associated factors in high fertile sub-Saharan Africa countries: a multi-level analysis of Demographic and Health Surveys

**DOI:** 10.1186/s13690-023-01210-3

**Published:** 2023-11-13

**Authors:** Tadele Biresaw Belachew, Desale Bihonegn Asmamaw, Ayele Biresaw Belachew, Mulatu Wubu Bayafers, Wubshet Debebe Negash

**Affiliations:** 1 Department of Health Systems and Policy, Institute of Public Health, College of Medicine and Health Sciences, University of Gondar, P.O.Box: 196, Gondar, Ethiopia; 2 Department of Reproductive Health, Institute of Public Health, College of Medicine and Health Sciences, University of Gondar, Gondar, Ethiopia; 3 University of Colorado Anschutz Medical Campus, Aurora, CO USA; 4 Ethiopian Public Health Institute, Addis Ababa, Ethiopia

**Keywords:** Decision-making autonomy, Modern contraceptive, Multilevel, Factors, Sub-Saharan Africa

## Abstract

**Background:**

For better maternal and child health, women’s independence on reproductive health issues is crucial; however, couples are restricted from discussing openly with their partner. Regarding this, information about women’s decision-making autonomy is low in the world, including Sub-Saharan Africa; therefore, this study was aimed to assess married women’s decision-making autonomy on modern contraceptive utilization in high fertility SSA countries.

**Methods:**

Data for this study was obtained from the most recent (2010–2018) Demographic and Health Surveys. A total of weighted sample of 14,575 married reproductive age women was included. A multilevel mixed-effect binary logistic regression model was fitted to identify the significant associated factors of decision-making autonomy on modern contraceptive utilization. Finally, the Adjusted Odds Ratio (AOR) with 95% confidence interval was used to declare as statistical significance.

**Results:**

Overall prevalence of married women decision-making autonomy on modern contraceptive utilization in the high fertile SSA countries is 25.28% (95% CI:18.32%, 32.24%). The factors significantly associated with the decision-making autonomy on modern contraceptive utilization were women’s age 25–34 years (AOR = 1.88, 95% CI = 1.84–1.93) and 35–49 years (AOR = 1.90, 95% CI = 1.82–1.92), had media exposure (AOR = 1.13, 95% CI = 1.00- 1.28), Number of alive children, 1–2 (AOR = 2.35, 95% CI = 1.38–4.01), 3–4 (AOR = 2.98, 95% CI = 1.74–5.10), $\ge$ 5 (AOR = 2. 82, 95% CI = 1.63–4.86), educational status; primary education (AOR = 1.93, 95% CI = 1.77–2.83), Secondary and higher (AOR = 2.11, 95% CI = 1.78–2.89), Community media exposure (AOR = 1.80, 95% CI = 1.38–2.34), Community level poverty, (AOR = 1.43, 95% CI = 1.09–1.86) and resides in rural (AOR = 0.67, 95% CI = 0.64–0.71).

**Conclusion:**

Women’s decision-making autonomy on modern contraception utilization in this study was low. Therefore, the government should promote women’s autonomy on contraceptive use as an essential component of SRH rights through mass media, with particular attention for, women living in the poorest communities, and those residing in rural settings of the country. Moreover, health professionals should counsel the women about the benefits of using modern contraceptive to help them managing their number of children.

**Table:** 

Text box 1. Contributions to the literature
➢ There is little information on how health system and policy affect married women’s autonomy in choosing a contraceptive in countries in Sub-Saharan Africa with high fertility rates.
➢ Information on the factors that help and hinder married women in sub-Saharan Africa make decisions about using contraceptives is necessary for public health strategies.
➢ By recognizing the empowerment of women in the decision-making process for contraceptive use, family planning activities in the area will be supported.

## Background

Decision-making autonomy refers to women’s ability to make their own choices in all matters related to their needs [1]. For better maternal and child health, women’s independence on reproductive health issues is crucial; however, couples are restricted from discussing openly about their family size due to gender-based restrictions [1]. An increase in women’s autonomy regarding health contributes to women’s empowerment [2]. A previous study found that women’s autonomy increased their use of contraceptives by 70% when they had greater control over their decision-making [3]. There was also evidence that the use of contraceptives was higher among women with decision-making autonomy [3–5]. Furthermore, the autonomy of women in decision-making is linked to other aspects of sexual and reproductive health (SRH), including unmet family planning needs [2], Unplanned pregnancy [6] as well as sexual behavior [7].

There have been studies that suggest women are less autonomous in their decision about contraception use and other SRH issues because of cultural influences and male dominance in the household [8, 9]. In developing countries, women are less likely to decide independently that they want to use contraception [2, 10, 11]. In 2017, 63% of married women worldwide used contraceptives, and 32% of women in Africa used these services [12].Studies in different parts of Africa have found that women’s decision-making autonomy on contraceptive use is one of the sociocultural factors affecting their uptake of contraceptive services [3, 7, 13–19]. However, low status of women due to deep-seated sociocultural barriers and gender norms prevents them from making decisions about their SRH issues on their own [20]. Across the globe, only 55% of married women have decision-making autonomy on SRH issues, with 36% in sub-Saharan Africa [21]. In Senegal, South Africa, and Ethiopia, about 6%, 41.3%, and 21.6% of married women decided to use contraception, respectively [2, 21, 22].

In previous studies, different community-level and individual-level factors have been identified that influence women’s decision-making autonomy regarding use of contraception. These include place of residence [23], age [10, 23–25], household wealth index [10], women’s education [11, 25–27] and occupation [10, 11, 23] women’s knowledge about contraceptive [24, 28, 29], religion [11], and number of living children [23].

Various strategies, policies, and programs have been implemented to improve safe motherhood at the global, regional, and national levels. Making their own educated decisions about sexual relations, the use of contraceptives, and the reproductive health care that was provided to women during the antenatal, delivery, and post-partum periods can help to increase the capacity of reproductive women in decision-making and exercising their sexual and reproductive health rights [30–32]. In addition, the Millennium Development Goals (MDGs) of 2000 subsequently supported the International Conference for Population Development (ICPD) recognition of SRH rights in 1994 and the 2030 Agenda for Sustainable Development Goals (SDGs) as part of the global movement toward universal health coverage in the 21st century [21, 33–35].

The findings from studies on women’s reproductive autonomy are still highly variable and inconclusive. Additionally, neither individual nor community-level factors influencing women’s decision-making autonomy on contraceptive use are being addressed in a way that will help policy-makers develop effective interventions based on the right evidence. Hence, this study was aimed to assess the level of married women’s decision-making autonomy on contraceptive use and its associated factors in top nine high fertility sub-Saharan Africa using a mixed-effect logistic regression model.

## Methods

### Study settings and data source

The study was pooled cross-sectional assessment of data from recent Demographic and Health Surveys (DHSs) conducted between January 2010 and December 2018 of ten countries in SSA. Angola, Burkina Faso, Burundi, Chad, Democratic Republic Congo, Gambia, Mali, Niger, and Nigeria, were included in this study. These countries were selected because they are the top nine countries with high fertility rates in SSA with fertility rates above 5.0, a higher value than the rate of 4.44 in SSA and 2.47 worldwide [36]. One country (Somalia) with no DHS data was excluded from the analysis. The data for these countries were obtained from the official database of the DHS program, www.measuredhs.com after authorization was granted via online request by explaining the purpose of our study. We used the woman-level dataset and extracted the dependent and independent variables. The DHS is a nationally representative household survey that uses face-to-face interviews on a wide range of population, health, nutrition tracking, and effect assessment measures. Study participants were selected using a two-stage stratified sampling technique. Enumeration Areas (EAs) were randomly selected in the first stage, while households were selected in the second stage. The analysis did not include women who did not use contraceptives, were not married or in a union and pregnant at the time of the survey. Thus, out of the total 159,014 reproductive age women included in the top nine high fertile SSA countries DHS, 16,979 pregnant women were excluded at an initial step and then 121,622 women who were not using contraceptives during the survey were dropped. Finally, women who were not in a union at the time of the survey were excluded which yielded an unweighted sample size of 6,609 women. We applied sampling weight to handle the disproportionate allocation of samples in the DHS and the final weighted sample size for this study was 14,575 (Table 1).

**Table 1:** Description of Surveys and sample size characteristics in high fertility countries in SSA (*n* = 14,575)

Countries	Survey year	Fertility rates	Weighted sample(n)	Weighted percentage (%)
Angola	2015	5.37	230	1.58
Burkina Faso	2010	5.03	1,973	13.54
Burundi	2016	5.24	2,055	14.10
Chad	2014	5.55	634	4.35
DR Congo	2013	5.72	1,687	11.57
Gambia	2013	5.09	567	3.89
Mali	2018	5.69	1,455	9.98
Nigeria	2012	5.25	4,606	31.60
Niger	2012	6.74	1,368	9.39

### Study variables

#### Outcome variable

The outcome variable of this study was married women’s decision-making autonomy on modern contraceptive use. For the purposes of the analysis, the outcome variable was divided into two categories: “not autonomous = 0” (for married reproductive age women who reported that the choice to use a contraceptive was primarily made by her husband/partner, respondent and her husband/partner, and others) and “autonomous = 1” (for married reproductive age women who reported that the choice to use a contraceptive was made only by themselves) [3, 26].

#### Explanatory variables

Both the individual and community level independent variables were included in this study.

Individual level variables; age, educational status of respondents, occupation, wealth status, media exposure, number of living children, husband education, and husband occupation.

Community level variables; the community level variables were residence and some were generated from the individual level data of all community members in primary sampling unit (PSU), which includes the community level poverty and community level media exposure.

### Data analysis

Stata version 16 software was used for data analysis. Throughout the analyses, sampling weight was used to adjust for the unequal probability of sample selection and the differences in response rates. The data were weighted to ensure the representativeness of the DHS sample and get reliable estimates and standard errors before data analysis.

Four models were fitted in this study: the null model, which had no explanatory variables, model I, which had individual-level factors, model II, which had community-level factors, and model III, which had both individual and community-level components. Since the models were nested, the Intra-class Correlation Coefficient (ICC), Median Odds Ratio (MOR) and, deviance (-2LLR) values were used for model comparison and fitness, respectively. Model III was the best-fitted model since it had the lowest deviance. Variables having a *p*-value less than 0.2 in bivariable analysis were used for multivariable analysis. Finally, in the multivariable analysis, adjusted odds ratios with 95% confidence intervals and a *p*-value of less than 0.05 were utilized to identify factors of Married Women’s decision-making autonomy on contraceptive use.

## Results

### Individual level factors

Approximately 45% of the women were aged between 25–34 years. Regarding their educational level, 40.43% women were reported with Secondary and above educational levels. Among the participants, 35.85% had more than five living children. Moreover, the majority of respondents 76.87% had media exposure. With regard to their economic status, 23.71% women were from the poor wealth quintiles and 59.09% were from the rich wealth quintiles (Table 2).

**Table 2:** Individual characteristics of respondents in high fertility countries in sub-Saharan Africa (*n* = 14,575)

Variables	Categories	Frequency	Percentage (%)
**Age of respondents**	15–24	2,342	16.07
	25–34	6,578	45.13
	35–49	5,655	38.80
**Educational status of respondents**	No formal education	5,320	36.50
	Primary education	3,362	23.07
	Secondary and above	5,893	40.43
**Husband education**	No formal education	4,881	33.52
	Primary education	2,775	19.06
	Secondary and higher	6,907	47.42
**Occupation of respondents**	Working	112,712	80.96
	Not working	2,651	19.04
**Husband occupation**	Employed	13,985	96.25
	Not employed	545	3.75
**Wealth index**	Poor	3,457	23.71
	Middle	2,506	17.20
	Rich	8,613	59.09
**Media exposure**	Yes	11,182	76.87
	No	3,365	23.13
**Number of alive children**	No	117	0.80
	1–2	4,217	28.93
	3–4	5,016	34.41
	$\ge$5	5,226	35.85

### Community level factors

Of the study participants more than half (55%) were resides in rural area. Above half (53.20%) of the reproductive age women were from communities with low proportion of poor households. More than half (52.49%) of participants had high community media exposure (Table 3).

**Table 3:** Community level characteristics of respondents in high fertility countries in sub-Saharan Africa (*n* = 14,575)

Variables	Categories	Frequency	Percentage (%)
**Residence**	Urban	6,549	44.93
	Rural	8,026	55.07
**Community-level poverty**	Low	7,754	53.20
	High	6,822	46.80
**Community media exposure**	Low	6,925	47.51
	High	7,651	52.49

### Prevalence of married women’s decision-making autonomy on modern contraceptive utilization

**Fig. 1:**
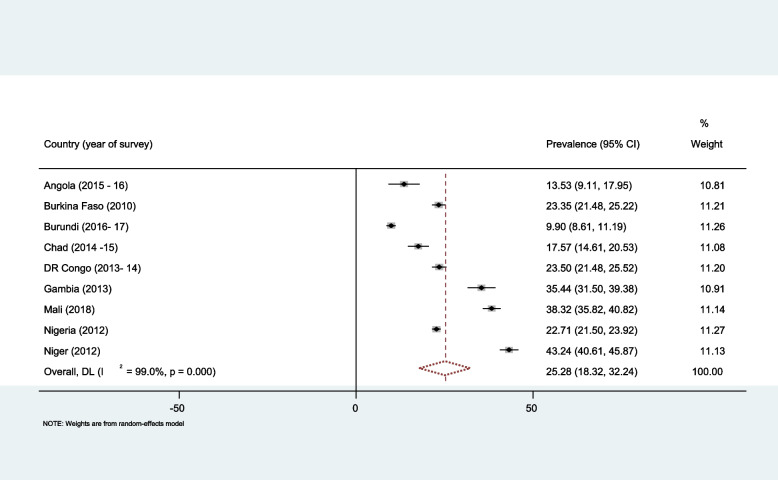
Forest plot of overall prevalence of married women decision making autonomy on contraceptive utilization in top nine high fertile Sub-Saharan countries. Source: Authors’ computations

Overall, the prevalence of decision-making autonomy on contraceptive use among married women in top nine high fertility sub-Saharan Africa was 25.28% (18.32%, 32.24%). The decision-making autonomy was ranged from 9.9% in Burundi to 43.24% in Niger (Fig. 1).

### Factors associated with married women’s decision-making autonomy on contraception utilization

In terms of the individual level factors, the study showed that reproductive age women aged 25–34 and 35–49 were more likely to have decision making autonomy [AOR = 1.88, 95% CI = 1.84–1.93] and [AOR = 1. 90, 95% CI = 1.82–1.92] respectively were compared with those aged 15–24. Married women who exposed for different types of media were 1.13 times decision making autonomous [AOR = 1.13, 95% CI = 1.00- 1.28] compared with non-exposed. The likelihood of married women’s decision-making autonomy contraceptive use was also higher among women who had 1–2 [AOR = 2.35, 95% CI = 1 S.38- 4.01], 3–4 [AOR = 2.98, 95% CI = 1.74–5.10] and ≥ 5 [AOR = 2. 82, 95% CI = 1.63–4.86] alive children compared to who did not have a child. Moreover, the decision-making autonomy contraception utilization was high among married women whose educational status primary education and secondary and higher were more likely [AOR = 1.93, 95% CI = 1.77–2.83], [AOR = 2.11, 95% CI = 1.78–2.89] respectively autonomous compared to those uneducated.

With regard to the community level factors, the odds of decision-making autonomy contraception utilization was higher among married women who had community media exposure [AOR = 1.80, 95% CI = 1.38–2.34] compared with unexposed. In addition, married women classified as low community level poverty ware more likely decision making autonomous [AOR = 1.43, 95% CI = 1.09–1.86] than high community level poverty. Married women who resides in rural were 67% less likely decision making autonomous [AOR = 0.67, 95% CI = 0.64–0.71] compared with urban (Table 4).

**Table 4:** Multivariable analyses for factors affecting married women’s decision making autonomy on contraceptive use (*n*** =** 14,575)

Variables	Model 0	Model 1 AOR (95% CI)	Model 2 AOR (95%CI)	Model 3 AOR (95%CI)
**Individual level Characteristics**				
Age				
15–24		Referent		Referent
25–34		1.14 (0.99–1.32)		1.88 (1.84–1.93)*
35–49		1.42 (1.19–1.67)		1. 90 (1.82 − 1.92)*
**Educational status of the respondents**				
No formal education		Referent		Referent
Primary education		0.99 (0.88–1.12)		1.93 (1.77–2.83)*
Secondary and higher		1.17 (1.05–1.32)		2.11 (1.78–2.89)*
**Wealth index**				
Poor		Referent		Referent
Middle		1.02 (0.88–1.17)		1.02 (0.88–1.18)
Rich		1.03 (0.91–1.17)		1.13 (0.98–1.29)
**Media exposure**				
No		Referent		Referent
Yes		1.11 (0.98–1.23)		1.13 (1.00- 1.28)*
**Number of alive children**				
No child		Referent		Referent
1–2		2.32 (1.36–3.94)		2.35 (1.38–4.01)*
3–4		2.95 (1.72–5.03)		2.98 (1.74–5.10)*
≥ 5		2.82 (1.64–4.86)		2. 82 (1.63–4.86)*
**Community level variables**				
** Community level poverty**				
High			Referent	Referent
Low			1.55 (1.18–2.03)	1.43 (1.09–1.86)*
** Community media exposure**				
Low			Referent	Referent
High			1.93 (1.47–2.52)	1.95 (1.38–2.34)*
**Residency**				
Urban			Referent	Referent
Rural			0.85 (0.82–0.88)	0.67 (0.64–0.71)*
**Random effect result**				
Variance (%)	4.54	3.76	2.85	2.12
ICC (%)	57.96	55.86	50.16	41.8
MOR	5.5	5.0	4.4	3.8
PCV	Ref	17.2%	37.2%	53.3%
Deviance(-2LLR)	145,494	135,106	145,316	134,734

Null model: adjusted for individual-level characteristics

Model 2: Adjusted for community-level characteristics

Model 3: adjusted for both individual and community level characteristics

*AOR* Adjusted Odds Ratio, *COR* Crude Odds Ratio

* Statistically significant at *p*-value < 0.05

## Discussion

Women’s decision making autonomy regarding contraception is one of the essential rights to sexual reproductive health [37]. In this study, we analyzed the levels of women’s autonomy and associated factors on contraceptive use in the top nine high fertile sub-Saharan countries from 2010 to 2018 DHS datasets. According to this finding, only 25.28% of women in top nine high fertility sub-Saharan countries have decision-making autonomy regarding contraception. This finding is in line with the previous studies in Mafikeng, South Africa [11], Nigeria [14], and Ethiopia [38, 39].

The prevalence of modern contraceptive decision making autonomy was, however, lower than those from other studies in Adwa, North Ethiopia [26], Dinsho, Southeast Ethiopia [24], rural districts of Southern Ethiopia [10], and South Africa [21] which reported that 36%, 52%, 58%, and 41%,of women respectively had decision-making autonomy on contraceptive use. Furthermore, the proportion of women in this study who had decision-making autonomy on contraception use was lower than those found in previous studies in Mizan-Aman 67% [27], Basoliben 80% [28], and Northwest Ethiopia 77% [40]. On the contrary, our finding is higher than a study in Senegal [2] which found that 6% of women had decision-making autonomy on contraceptive use. Perhaps the discrepancy is due to the differences in methodology of the studies.

Multivariable multilevel logistic regression model identified the place of residence, community level poverty, community level media exposure, educational status of respondent, exposure to mass media, current age, and number of children as the factors affecting women’s decision-making autonomy on contraceptive use. Accordingly, women from rural dwellings had a lower likelihood of having decision-making autonomy on contraceptive use compared to those residing in urban settings. This finding is consistent with the result of the previous studies in Ethiopia [2, 29] which found higher decision-making autonomy on contraceptive use among urban women. The reason may be that urban women have better access to education and information about contraceptives and other SRH-related issues than their rural counterparts do, which enables them to influence household decision-making and choose contraception.

Women in a community with a high exposure to family planning messages were nearly twice more likely to be decision-making autonomy on contraceptive use than their counter counterparts. This finding is in line with the result of a study done in Ethiopia [38]. Furthermore, it is supported by the finding of a study in Pakistan that reported a positive relationship between women’s decision-making autonomy and their awareness about family planning [41].Regarding community level poverty, economic resources can inhibit the ability of women to decide on their human and reproductive rights including contraceptive decision-making. Likewise, in this study, the low level of community poverty had High likelihood of women decision-making to use contraceptives as compared to those who found in the high-level community poverty class.

The odds of decision-making autonomy on contraceptive use among women aged 25–34 years, 35–49 years were 1.88 and 1.90 times higher compared to those women from the age of 15–24 years respectively. It is similar to previous studies conducted in Ethiopia [2, 10, 23], which was reported higher decision-making autonomy on contraceptive use as the age of respondents increased. The reason for this is that younger women are less likely to attend family planning clinics and are unaware of SRH due to limited access to information about SRH [42], and younger women may be trying to start a family and therefore less likely to use modern methods. In addition, women may not be exposed to modern contraceptive methods until they have been exposed to the health care system (community health workers, nurses, midwives) due to pregnancy and childbirth. Due to this, they have limited control over their contraceptive decision.

Women’s decision-making autonomy for using contraceptives was significantly more likely among women who attended primary, secondary and above education compared that of non-educated. This is consistent with the previous studies [43, 44]. The possible justification is, education empowers women to be independent and equips them with the essential information that may important for deciding their reproductive health issues. Additionally, educated women participate in collective decision-making with their husbands regarding their health, children’s health, and visits to relatives or family members, which facilitates sharing experiences and exercising their reproductive and human rights. Likewise, other previous studies [16, 27, 45–47] showed that the more educated the women and their partners, the more likely they were to decide on family planning use.

Women who have access to sources of contraceptive information are better able to make informed choices regarding fertility control services and are able to determine whether these services will work for them. Accordingly, Women exposed to mass media increased their decision-making autonomy on contraceptive use by 1.13 comparing to those who did not, which was supported by the positive relationship between the use of contraceptive methods and exposure to mass media.

Based on this study, having one or more children increases the likelihood that a woman’s decision-making autonomy to use modern contraceptives as compared to not having children. According to the 2011 Ethiopian Demographic and Health Survey (EDHS) data analysis [48] and a study done in the Southern Nations Nationalities and Peoples’ Region (SNNPR) [49] researchers also found that an increase in the number of living children was significantly associated with an increase in woman’s decision-making autonomy on modern contraceptive utilization.

In this study, the cluster effect was handled with an advanced mixed-effects model, which used nationally representative data. However, the cross-sectional design of the DHS data used in this study, which did not show cause and effect relationship to explanatory and outcome variable. In addition, information about women’s decision-making autonomy on contraceptive use was collected based on self-reporting, which is likely to be subjected to recall and social desirability bias.

## Conclusions

Married women’s decision-making autonomy on modern contraception utilization in this study was low. This study showed that place of residence, community exposure to mass media, Community level poverty, women’s age, educational status of respondents, media exposure, and numbers of children were identified as the factors affecting women’s decision-making autonomy on contraceptive use.

Therefore, the government should promote women’s autonomy on contraceptive use as an essential component of SRH rights through mass media, with particular attention for, women living in the poorest households, and those residing in rural settings of the country. Moreover, health professionals should counsel the women about the benefits of using modern contraceptive to help them managing their number of children.

## Data Availability

Data for this study were sourced from Demographic and Health surveys (DHS), which is freely available online at (https://dhsprogram.com).

## References

[1] Mistry R, Galal O, Lu M (2009). Women’s autonomy and pregnancy care in rural India: a contextual analysis. Soc Sci Med.

[2] Sougou N, Bassoum O, Faye A, Leye M (2020). Women’s autonomy in health decision-making and its effect on access to family planning services in Senegal in 2017: a propensity score analysis. BMC Public Health.

[3] OlaOlorun FM, Hindin MJ (2014). Having a say matters: influence of decision-making power on contraceptive use among Nigerian women ages 35–49 years. PLoS One.

[4] Viswan SP, Ravindran TS, Kandala N-B, Petzold MG, Fonn S (2017). Sexual autonomy and contraceptive use among women in Nigeria: findings from the demographic and health survey data. Int J Womens Health.

[5] Wado YD. Women’s autonomy and reproductive healthcare-seeking behavior in Ethiopia. ICF International; 2013.

[6] Kassahun EA, Zeleke LB, Dessie AA, Gersa BG, Oumer HI, Derseh HA (2019). Factors associated with unintended pregnancy among women attending antenatal care in Maichew Town, Northern Ethiopia, 2017. BMC Res Notes.

[7] Biswas AK, Shovo TEA, Aich M, Mondal S (2017). Women’s autonomy and control to exercise reproductive rights: a sociological study from rural Bangladesh. SAGE Open.

[8] Andrzej K (2008). Husband-wife agreement, power relations and contraceptive use. Int Fam Plan Perspect.

[9] Santelli JS, Lindberg LD, Orr MG, Finer LB, Speizer I (2009). Toward a multidimensional measure of pregnancy intentions: evidence from the United States. Stud Fam Plann.

[10] Alemayehu M, Meskele M (2017). Health care decision making autonomy of women from rural districts of Southern Ethiopia: a community based cross-sectional study. Int J Womens Health.

[11] Osuafor GN, Maputle SM, Ayiga N (2018). Factors related to married or cohabiting women’s decision to use modern contraceptive methods in Mahikeng, South Africa. Afr J Prim Health Care Fam Med.

[12] Kantorová V, Wheldon MC, Ueffing P, Dasgupta AN (2020). Estimating progress towards meeting women’s contraceptive needs in 185 countries: a bayesian hierarchical modelling study. PLoS Med.

[13] Loll D, Fleming PJ, Manu A, Morhe E, Stephenson R, King EJ (2019). Reproductive autonomy and modern contraceptive use at last sex among young women in Ghana. Int Perspect Sex Reprod Health.

[14] Alabi O, Odimegwu CO, De-Wet N, Akinyemi JO (2019). Does female autonomy affect contraceptive use among women in northern Nigeria?. Afr J Reprod Health.

[15] Adhikari R, Acharya D, Ranabhat CL, Ranju K (2019). Factors associated with non-use of contraceptives among married women in Nepal. J Health Promot.

[16] Tadesse M, Teklie H, Yazew G, Gebreselassie T. Women’s empowerment as a determinant of contraceptive use in Ethiopia further analysis of the 2011 Ethiopia demographic and health survey. DHS Furth Anal Rep. 2013;82.

[17] Al Riyami A, Afifi M, Mabry RM (2004). Women’s autonomy, education and employment in Oman and their influence on contraceptive use. Reprod Health Matters.

[18] Atiglo DY, Codjoe SN (2019). Meeting women’s demand for contraceptives in Ghana: does autonomy matter?. Women Health.

[19] Rahman MM, Mostofa MG, Hoque MA (2014). Women’s household decision-making autonomy and contraceptive behavior among Bangladeshi women. Sex Reprod Healthc.

[20] Peters JS, Wolper A. Women's rights, human rights: international feminist perspectives. Routledge; 2018.

[21] Mare KU, Aychiluhm SB, Tadesse AW, Abdu M (2022). Married women’s decision-making autonomy on contraceptive use and its associated factors in Ethiopia: a multilevel analysis of 2016 demographic and health survey. SAGE Open Med.

[22] Africa Dis. The routledge handbook of disability in Southern Africa.

[23] Edossa ZK, Debela TF, Mizana BA (2020). Women’s decision on contraceptive use in Ethiopia: multinomial analysis of evidence from Ethiopian demographic and health survey. Health Serv Res Manag Epidemiol.

[24] Dadi D, Bogale D, Minda Z, Megersa S (2020). Decision-making power of married women on family planning use and associated factors in Dinsho Woreda, South East Ethiopia. Open Access J Contracept.

[25] Osamor PE, Grady C (2016). Women’s autonomy in health care decision-making in developing countries: a synthesis of the literature. Int J Womens Health.

[26] Alemayehu M, Hailesellasie K, Biruh G, Gebrezgabiher G, Tinsae F, Kidanemariam A (2014). Married women’s autonomy and associated factors on modern contraceptive use in Adwa Town, Northern Ethiopia. Science.

[27] Belay AD, Mengesha ZB, Woldegebriel MK, Gelaw YA (2016). Married women’s decision making power on family planning use and associated factors in Mizan-Aman, South Ethiopia: a cross sectional study. BMC Womens Health.

[28] Alemayehu B, Kassa GM, Teka Y, Zeleke LB, Abajobir AA, Alemu AA (2020). Married women’s decision-making power in family planning use and its determinants in Basoliben, Northwest Ethiopia. Open Access J Contracept.

[29] Bogale B, Wondafrash M, Tilahun T, Girma E (2011). Married women’s decision making power on modern contraceptive use in urban and rural southern Ethiopia. BMC Public Health.

[30] Rammohan A, Johar M (2009). The determinants of married women’s autonomy in Indonesia. Fem Econ.

[31] Kebede AA, Cherkos EA, Taye EB, Eriku GA, Taye BT, Chanie WF (2021). Married women’s decision-making autonomy in the household and maternal and neonatal healthcare utilization and associated factors in Debretabor, northwest Ethiopia. PLoS One.

[32] Acharya DR, Bell JS, Simkhada P, Van Teijlingen ER, Regmi PR (2010). Women’s autonomy in household decision-making: a demographic study in Nepal. Reprod Health.

[33] Akwara E, Idele P (2020). The moral and social narratives of sexual and reproductive health in Kenya: a case of adolescents and young people pre-and within the MDG era. Reprod Health.

[34] Organization WH. Annual technical report 2015: department of reproductive health and research, including UNDP/UNFPA/WHO/World bank special programme of research training in Human Reproduction (HRP). World Health Organization; 2016.

[35] Toure K, Presern C (2016). Positioning women’s and children’s health in the post-2015 sustainable development agenda. Pathways Global Health.

[36] African countries with the highest fertility rate | Statista https://worldpopulationreview.com/countries/total-fertility-rate. Cited on December 8, 2021.

[37] Kanem N (2018). Sexual and reproductive health and rights: the cornerstone of sustainable development. UN Chron.

[38] Delbiso TD (2013). Gender power relations in reproductive decision-making: the case of Gamo migrants in Addis Ababa, Ethiopia. Afr Popul Stud.

[39] Eshete A, Adissu Y (2017). Women’s joint decision on contraceptive use in Gedeo zone, Southern Ethiopia: a community based comparative cross-sectional study. Int J Fam Med.

[40] Yonas Tadesse S, Emiru AA, Tafere TE, Asresie MB (2019). Women’s autonomy decision making power on postpartum modern contraceptive use and associated factors in north west Ethiopia. Adv Public Health.

[41] Nadeem M, Malik MI, Anwar M, Khurram S (2021). Women decision making autonomy as a facilitating factor for contraceptive use for family planning in Pakistan. Soc Indic Res.

[42] Rios-Zertuche D, Blanco LC, Zúñiga-Brenes P, Palmisano EB, Colombara DV, Mokdad AH (2017). Contraceptive knowledge and use among women living in the poorest areas of five mesoamerican countries. Contraception.

[43] Darteh EKM, Doku DT, Esia-Donkoh K (2014). Reproductive health decision making among Ghanaian women. Reprod Health.

[44] Sujatha DS, Reddy GB (2009). Women’s education, autonomy, and fertility behaviour. Asia Pac J Soc Sci.

[45] Kinoshita R. Women’s domestic decision-making power and contraceptive use in rural Malawi. Reprod Health. 2003;2(8).

[46] Erci B (2003). Women’s efficiency in decision making and their perception of their status in the family. Public Health Nurs.

[47] Kebede Y (2006). Contraceptive prevalence in Dembia district, northwest Ethiopia. Ethiop J Health Dev.

[48] Lakew Y, Reda AA, Tamene H, Benedict S, Deribe K (2013). Geographical variation and factors influencing modern contraceptive use among married women in Ethiopia: evidence from a national population based survey. Reprod Health.

[49] Endriyas M, Eshete A, Mekonnen E, Misganaw T, Shiferaw M, Ayele S (2017). Contraceptive utilization and associated factors among women of reproductive age group in Southern Nations nationalities and peoples’ region, Ethiopia: cross-sectional survey, mixed-methods. Contracept Reprod Med.

